# How to Get a Gold or Brass Ring from the Finger

**Published:** 1874-10

**Authors:** William Hauser

**Affiliations:** Bartow, Ga.


### HOW TO GET A GOLD OR A BRASS RING FROM THE FINGER.

By WILLIAM HAUSER, M.D., of Bartow, Ga.

Many years ago I learned from a young Charleston doctor, whose name I never knew, how to get a gold ring from a finger that had swollen too much to admit of its removal in the ordinary way. But the *how* to get off a *brass* ring, under similar circumstances, is my own discovery—a discovery with me, though thousands of others may, for aught I know to the contrary, have made the same.

I had a quiz-class in the Richmond (Va.) Medical College, one session, during the war, and to them I propounded the question as to getting off a gold ring under the circumstances named above. All failed to answer it, and I told them the Charleston doctor's plan. He was sent for on an occasion by an old physician, after he himself had failed, to remove a ring from the finger of a fat young girl, the old doctor paying him the compliment to tell him he had sent for him on account of his known mechanical skill. But the young doctor, fortunately, knew chemistry as well as mechanics, and this knowledge saved the finger, which otherwise had been doomed to amputation. Knowing the intense affinity of gold and quicksilver for each other, he, after having wiped the finger perfectly dry, poured the quicksilver over the ring; a chemical union was instantly formed, and, taking the ring between his thumb and fore-finger, by a sudden pressure he snapped it into pieces. After I had told all this, one of the students begged leave, very respectfully, to ask me how I would treat a brass ring similarly situated. I gave it up. The class appealed to Dr. James B. McCaw, Professor of Chemistry. He gave it up. I asked him about it myself afterward, but, like myself, he could not think how to do it. To-day, after the lapse of so many years, I providentially discovered the *how* in this case, and here it is, "as plain as a pike-staff:"

Pour melted white wax, or bees-wax, all over the ring, enough to fill up all the gaps between the ridges on the afflicted finger; make it run under the ring also, to insure full protection to the skin; then, with a pen-knife, cut out a small hole in the wax down to the ring; then pour this hole full of sulphuric, nitric, or muriatic acid. Let this stand until the copper and zinc (of which brass is composed) are combined with the acid. The ring can then be broken off by a pressure of the fingers. If acid sulph. be used, it will form with the ring the sulphates of copper and of zinc.

